# The Role of Food Insecurity in Relation to Meals on Wheels Participation

**DOI:** 10.1093/geroni/igaf122.477

**Published:** 2025-12-31

**Authors:** Laura Samuel, Emily Gadbois, Kimberly Bernard, Claire Wang, Nichole Stetten, Tiffany Riser, Christopher Liu, Kali Thomas

**Affiliations:** Johns Hopkins University, Baltimore, Maryland, United States; Brown University School of Public Health, Providence, Rhode Island, United States; Brown University, Providence, Rhode Island, United States; Johns Hopkins University, Baltimore, Maryland, United States; Brown University, Providence, Rhode Island, United States; Johns Hopkins University, Baltimore, Maryland, United States; Brown University, Providence, Rhode Island, United States; Johns Hopkins University, Baltimore, Maryland, United States

## Abstract

Deliver-EE participants have a five-fold higher risk of food insecurity than typical U.S. older adults (47% vs. 9% based on 2023 national data), defined as the inability to afford a consistently balanced diet. This is notable because food insecurity is associated with adverse outcomes including health care spending and dementia risk. Based on subgroup data (n = 376), 93% of participants have financial strain where they had either “just enough” or “not enough” money to make ends meet. This study explored the reasons for enrolling in Meals on Wheels using semi-structured interviews among 31 participants experiencing food insecurity or financial strain. Preliminary findings found many enrolled because they couldn’t afford food and/or transportation, as evidenced by one participant, “I just didn’t have the money.” Others described how financial strain in the presence of functional difficulties with shopping and/or cooking led them to seek meals, including one who said “So it’s kind of hard to get out and shop. Plus, we’re both low income, and we’re seniors.”. Notably, 37% with food insecurity had Activities in Daily Living (ADL) limitations and 72% had Instrumental ADL limitations, highlighting the co-occurrence of food insecurity and functional limitations. Participants also discussed that prepared foods are typically more expensive and less nutritious than food cooked at home; many said that they enrolled because they wanted a balanced and affordable diet that included hot food. These results suggest that Meals on Wheels meets a critical nutrition gap for food insecure older adults, especially those with functional limitations.

